# The Full Burden of RSV-related Pediatric Intensive Care Unit Admissions During Infancy: A Prospective National Study (BRICK Study)

**DOI:** 10.1097/INF.0000000000004712

**Published:** 2025-03-04

**Authors:** Emily W.E.M. Phijffer, Joanne G. Wildenbeest, Carole N.M. Brouwer, Matthijs de Hoog, Martin C.J. Kneyber, Sofie Maebe, Anneliese Nusmeier, Maaike A. Riedijk, Roelie M. Wösten-van Asperen, Louis J. Bont, Job B.M. van Woensel

**Affiliations:** From the * Department of Paediatric Infectious Diseases and Immunology; †† Paediatric Intensive Care, Wilhelmina Children’s Hospital, University Medical Center, Utrecht, the Netherlands; † Paediatric Intensive Care, Leiden University Medical Centre, Leiden, the Netherlands; ‡ Division of Paediatric Intensive Care, Department of Neonatal and Paediatric Intensive Care, Erasmus MC-Sophia Children’s Hospital, Rotterdam, the Netherlands; § Division of Paediatric Critical Care Medicine, Department of Paediatrics, Beatrix Children’s Hospital, University Medical Centre Groningen, Groningen, the Netherlands; ¶ Paediatric Intensive Care, Maastricht University Medical Centre, Maastricht, the Netherlands; ‖ Paediatric Intensive Care, Amalia Children’s Hospital, Radboud University Medical Centre, Nijmegen, the Netherlands; ** Paediatric Intensive Care Unit, Emma Children’s Hospital, Amsterdam UMC location University of Amsterdam, Amsterdam, the Netherlands.

**Keywords:** respiratory syncytial viruses, bronchiolitis, infant, critical care, tertiary care centers

## Abstract

**Background:**

Worldwide, respiratory syncytial virus (RSV) is the leading cause of lower respiratory tract infections during infancy. Approximately 5% of hospitalized infants require pediatric intensive care unit (PICU) admission. Our objective was to capture a complete overview of the PICU admission, including the yet unseen burden, to understand the full clinical impact of new preventive interventions.

**Methods:**

A nationwide, prospective, observational, multicenter study was performed in the Netherlands. Patients included infants <12 months with laboratory-confirmed RSV-related PICU admission. Collected data were clinical characteristics, unwanted and detrimental events during admission and parental signs of post-traumatic stress disorder (PTSD) after admission.

**Results:**

Of 423 patients, most were term born (n=335, 79.2%) and had no comorbidities (n=292, 69.0%). Median age was 46 (interquartile range, 25.0–89.0) days. Invasive mechanical ventilation was used in 258 (61.1%) patients. In total, 51 unwanted events were observed in 48 (11.3%) patients. Events occurred more frequently in patients receiving invasive mechanical ventilation compared to patients receiving other types of respiratory support (91.9% versus 52.4%; *P* < 0.001). Three (0.7%) patients died, all with severe comorbidities. Follow-up showed signs of PTSD in 35/120 (29.2%) of the parents.

**Conclusions:**

The burden of severe RSV disease is broader than the PICU admission alone. During admission, 11% of infants experienced unwanted, detrimental events, and one-third of parents showed signs or symptoms of PTSD. Because 75% of infants were <3 months of age, the introduction of RSV immunization into the Dutch immunization program could have the potential to significantly decrease this yet unseen RSV burden too.

Worldwide, respiratory syncytial virus (RSV) is the leading cause of life-threatening lower respiratory tract infections (LRTIs) during infancy. Annually, it is estimated that RSV infection results in 33.3 million episodes of acute LRTIs, 3.6 million hospital admissions and 26,300 in-hospital deaths worldwide.^1^ Approximately 5% of hospitalized infants experience severe RSV-LRTI and require pediatric intensive care unit (PICU) admission,^2^ which is a stressful event for patients and families.^3^

Most infants admitted to the PICU are below 3 months of age.^2^ Over the last 2 decades, RSV-related PICU admissions have increased in children <2 years of age in Europe.^4^ In the Netherlands, a 4-fold increase in RSV-related PICU admissions was observed between 2003 and 2016 (from 13.5 to 48.0 per 100,000 infants <24 months).^5^ This was mostly driven by an increase in PICU admissions of children below 3 months of age.^5^ Each year, during the RSV season, the available PICU capacity is stretched to the limit.

RSV preventive interventions could have a profound impact on RSV-related PICU admissions.^6,7^ An extended half-life monoclonal antibody (nirsevimab) showed an efficacy of 77.3% (95% confidence interval, 50.3–89.7) in preventing RSV-related hospitalizations in healthy preterm and term infants.^8^ Recently, nirsevimab was approved by the US Food and Drug Administration and the European Medicines Agency for use in infants.^9,10^ In addition, the US Food and Drug Administration and European Medicines Agency approved a maternal RSV vaccine,^11,12^ which showed a 67.7% vaccine efficacy (99.17% confidence interval, 15.9–89.5) in preventing hospitalization due to RSV within 90 days after birth in their phase 3 trial.^13^ We previously investigated the health-economic impact of vaccination and found that the introduction of both new RSV preventive interventions into the Dutch immunization program will generate significant cost-savings for PICUs.^14^ The estimated potential costs averted are € 1.9 to € 2.6 million depending on the season and the duration of protection. The Dutch Health Council advised to implement the monoclonal antibody in the Dutch immunization program. The implementation is scheduled for the autumn of 2025.^15^

To understand the full clinical impact of RSV prevention, insight into the yet unseen burden of severe RSV disease will be helpful. Therefore, this study aims to capture a complete overview of PICU admission, including unwanted, detrimental events during PICU admission and long-term parental psychologic sequelae after PICU admission.

## METHODS

### Design

A nationwide, prospective, observational, multicenter study was performed from September 2021 to June 2023 at all 7 PICUs in the Netherlands. The total number of operational PICU beds in the Netherlands is, dependent on nurse availability, up to 100.^16^

### Study Population

The BRICK (Burden of RSV-Related Intensive Care Admissions of Kids) study was initiated with the aim to collect data during 2 RSV seasons (2021–2022 and 2022–2023). Because of continuous RSV circulation, data collection for the first season was extended until September 2022 and was directly followed by the second season, starting in October 2022 and ending in mid-June 2023. Clinicians of all Dutch PICUs were asked to share clinical data of all RSV-related PICU admissions of infants ≤12 months of age. An RSV-related PICU admission was defined as a PICU admission of ≥1 day with a laboratory-confirmed RSV infection. Clinical data were collected up to 1 year after PICU admission.

### Data Collection

The study collaborators shared data on newly admitted patients weekly. To ensure complete data collection, the electronic health record (EHR) of all included patients was checked by the research team regularly. Duplicates resulting from readmissions for the same disease episode and/or transfers between PICUs were removed. All data were entered into Castor EDC.^17^ The following data were obtained from the EHRs: age at PICU admission, gender, gestational age at birth, comorbidities (including prematurity, defined as <37 weeks of gestation), bronchopulmonary dysplasia, congenital heart disease and Down syndrome, type of most invasive respiratory support, duration of respiratory support, PICU length of stay (LoS) and results of virology testing.

In addition, we obtained prespecified unwanted, detrimental events occurring during the course of PICU admission and long-term psychological sequelae after PICU discharge. Unwanted, detrimental events during the course of PICU admission included the following:

- reintubation due to ongoing respiratory distress after the attempt to extubate the patient;
- intubation injuries (confirmed subglottic stenosis) after intubation of the patient;
- pneumothorax;
- bronchospastic events resulting in resuscitation (CPR);
- bradycardia resulting in CPR;
- convulsions;
- extravasation of infusion fluid.

The occurrence of events was checked in the hospital discharge letter, and if necessary, additional information about the event was retrieved from the EHR. In case reported events were unclear, E.W.E.M.P. and J.B.M.v.W. reviewed the event independently. If needed, a third reviewer was consulted (J.B.M.v.W.), and events were included after consensus was reached.

Long-term parental psychologic sequelae were evaluated during routine follow-up visits 3-6 months after PICU discharge. In the Netherlands, follow-up care of PICU-admitted patients is the standard of care and is in line with the national guideline on follow-up.^18^ Part of this follow-up is the evaluation of the psychosocial outcomes in parents. For this study, we collected information from domain 2 including parental signs or symptoms of post-traumatic stress disorder (PTSD). Parental signs or symptoms of PTSD were assessed by the pediatric intensive care specialist and/or a psychologist using a validated psychological questionnaire.

The validated questionnaire for PTSD, called PTSD checklist for DSM-5, may differ slightly in each hospital, yet it is based on the Diagnostic and Statistical Manual of Mental Disorders criteria^19^ and was previously used to investigate PTSD and other physiological outcomes in parents from children with multisystem inflammatory syndrome after PICU admission in the Netherlands.^20^ The questionnaire includes 20 items that reflect the DSM-5 diagnostic criteria of PTSD. Parents were asked to self-report each item on a Likert scale ranging from 0 to 4. A higher score represents more pronounced PTSD signs or symptoms. Results were reported to the EHR by the pediatric intensive care specialist and/or a psychologist after the follow-up visit.

The EHRs were checked up to 1 year after discharge to ensure that the follow-up visit was completed. All parents who completed follow-up with data about the occurrence of signs or symptoms of PTSD were included in the PTSD analysis. In case no data were available, they were left out of the analysis. Parents were reported as positive for signs or symptoms of PTSD in case they: (1) scored positive for signs of PTSD on a validated psychological questionnaire used during the follow-up visit or/and (2) reported treatment for signs or symptoms of PTSD elsewhere.

### Statistical Analyses

Summary estimates were expressed as proportions and median numbers with interquartile range (IQR). Baseline characteristics and clinical parameters were compared between RSV seasons and between the administered types of respiratory support using χ^2^ tests for categorical variables and Mann-Whitney *U* tests for not normally distributed continuous variables. Statistical analyses were performed using SPSS, version 27.0.

## RESULTS

### Baseline Characteristics

A total of 424 patients with an RSV-related PICU admission were identified. Of these 424 patients, 423 patients with available clinical data were included in this analysis. Baseline characteristics are shown in Table 1. The median age at PICU admission was 46 (IQR, 25.0–89.0) days. In total, 320 of the 423 patients (75.7%) were younger than 3 months of age on PICU admission Most patients were born term (n=335, 79.2%) and did not have comorbidities (n=292, 69.0%). Characteristics did not differ between seasons.

**Table 1.** Descriptive Characteristics of RSV-Related PICU-Admitted Infants

Characteristic	PICU-admitted infants (n=423)*
Age at PICU admission (median days, IQR)	46.0 (25.0–89.0)
Gender (male), n (%)	238 (56.3%)
Comorbidities, n (%)	131 (31%)
CHD	29 (6.9%)
Prematurity	88 (20.8%)
DS	8 (1.9%)
BPD	3 (0.7%)
Other comorbidities^†^	46 (10.9%)
LoS (median days, IQR)	5.0 (3.0–8.0)

* Data of 1 patient in season 1 was missing.

† Other comorbidities include 7q11.23 deletion, *CTFT* gene mutation, congenital disorders of glycosylation type 1w, cutis aplasia congenita, erythrocyte membrane defect, ectopic kidney, thyroid agenesis, laryngo(tracheobroncho)malacy, cystic kidney disease, periventricular leukomalacia, anorectal malformation, suspected Russells syndrome, Emanuel syndrome, dysplastic monokidney, dolichosigmoid, iron deficiency anemia, hypospadia, horseshoe kidney (*PRKD1* variant), G6PD deficiency, hypotonia e.c.i., jejunal atresia, hand hypoplasia, hemangioma, hernia diaphragmatica, hernia umbilicalis, hernia inguinalis, intraventricular hemorrhage, malrotation and annular pancreas, Mb deletion of 19p13, undescended testes, DPD deficiency, lung disease immuno-deficiency and chromosome breakage syndrome, lymphatic malformation, trisomy X, trisomy 16 with iris coloboma and immature bowel, MCAD deficiency, Pierre Robin sequence, perinatal asphyxia, persistent pulmonary hypertension, portal vein thrombosis, pyloric hypertrophy, *RARA* gene variant, neonatal brachial plexus palsy, SMA type 2, West syndrome, XXYY syndrome and pes equinovarus.

BPD indicates bronchopulmonary disease; CHD, congenital heart disease; DS, down syndrome; DPD, dihydropyrimidine dehydrogenase; ECMO, extracorporeal membrane oxygenation; G6PD, glucose-6-phosphate dehydrogenase; MCAD, medium-chain acyl-CoA dehydrogenase; n, number of infants; SMA, spinal muscular atrophy.

### Course and Events in RSV-Related PICU Admissions

The median length of PICU admission was 5 days (IQR, 3.0–8.0). In 258/423 (61.1%) patients, invasive mechanical ventilation (IMV) was used, and 164/423 (38.9%) received other modes of respiratory support including high-flow nasal cannula (HFNC; Table 2). The median duration of IMV was 5 (IQR, 4.0–7.5) days. The PICU LoS was higher in patients receiving IMV compared with patients receiving other types of respiratory support (median days, 7.0 versus 3.0; *P* < 0.001). In addition, IMV was more often used in patients born prematurely compared to term-born infants (71.6% versus 58.4%; *P* = 0.027).

**Table 2.** Maximum Respiratory Support in PICU-Admitted Infants

Characteristic	PICU-admitted infants (n=423)*
Invasive ventilation, n (%)	258 (61.1%)
Duration (median days, IQR)	5.0 (4.0–7.5)
Noninvasive ventilation, n (%)	62 (14.7%)
Duration (median days, IQR)	2.0 (1.0–3.0)
HFNC, n (%)	93 (22.0%)
Duration (median days, IQR)	2.0 (1.0–2.0)
Low-flow support, n (%)	9 (2.1%)
Duration (median days, IQR)	1.0^†^
ECMO, n (%)	0 (0.0%)

* Data of 1 patient in season 1 was missing. One infant did not receive respiratory support.

† Eight infants were 1 day supported with low flow, and 1 infant was 2 days supported with low flow.

ECMO indicates extracorporeal membrane oxygenation; n, number of infants.

During the course of the PICU admission, a total of 51 unwanted, detrimental events were observed in 48 (11.3%) patients of which most frequently observed were reintubation after extubation (n=12, 2.8%), intubation injuries (n=11, 2.6%), and extravasation of infusion fluids (n=12, 2.8%).

All unwanted, detrimental events are shown in Table 3. Forty-five patients (10.6%) suffered from 1 event, and 3 patients (0.7%) had 2 events. Events occurred in 47/258 (18.2%) of patients receiving IMV and in 1164 (0.6%) of patients receiving other types of respiratory support (*P* ≤ 0.001). Three (0.7%) patients died during PICU admission, all with severe comorbidities (Table 4).

**Table 3.** Severe Events During RSV-Related PICU Admission

Event	Intervention	Total (n=423)*
Total		48 (11.3%)
Ongoing respiratory distress after extubation	Reintubation	12 (2.8%)
Intubation injury (confirmed subglottic stenosis)	Reintubation and dilatation/surgery	3 (0.7%)
	Dilatation/surgery	5 (1.2%)
	Dexamethasone and antibiotics and/or esomeprazole	2 (0.5%)
	None	1 (0.2%)
Pneumothorax	Tube drainage	6 (1.4%)
Bronchospastic events	CPR	4 (0.9%)
Bradycardia	CPR	1 (0.2%)
Convulsions	Midazolam i.v.	2 (0.5%)
Infusion fluid extravasation	Hyaluronidase/phentolamine injection	4 (0.9%)
	Supportive care (eg, switch positioning and/or thermal skin therapy)	8 (1.9%)
Death		3 (0.7%)

* Data of 1 patient in season 1 was missing. n (%) numbers of infants per event are not mutually exclusive, and infants can have multiple events in each category.

**Table 4.** Descriptive Characteristics of Infant Deaths During RSV-Related PICU Admission

Infant	Description
1	Patient 1 was 4 months of age on admission, was born prematurely (GA, 27 weeks) with Down syndrome and suffered from a hemodynamically significant congenital heart disease.
2	Patient 2 was 5 months of age on admission, was born prematurely (GA, 35 weeks), had several comorbidities including a congenital heart disease due to a genetic abnormality and developed necrotizing enterocolitis.
3	Patient 3 was 11 months at admission, was born term, had trisomy X and dihydropyrimidine dehydrogenase deficiency and was diagnosed with a lethal lung disease during RSV-related PICU admission.

GA indicates gestational age.

### Readmission

In total, 18/423 (4.2%) patients required readmission to the PICU after discharge, which was related to the initial PICU admission. Reasons for readmission were a biphasic course of the RSV infection in 13/18 patients (72.2%), persistent severe upper airway obstruction in 4/18 (22.2%) for which 2 patients required surgery, and necrotizing enterocolitis in 1/18 (5.6%) patient, which occurred during the course of RSV infection.

### Long-Term Sequelae After Discharge

Psychologic assessment was performed during 120/167 (71.9%) outpatient clinic visits. A total of 35 (29.2%) of these parents showed signs or symptoms of PTSD.

## DISCUSSION

To our knowledge, this is the largest national, prospective, observational, multicenter study evaluating the course of PICU admission including unwanted events. Our study demonstrates that the burden of severe RSV disease is broader than the PICU admission alone. We found that ≈11% of infants admitted to the PICU for RSV infection experienced unwanted, detrimental events during PICU admission, such as intubation injuries.

Few other studies, largely based on case series, described events occurring during PICU admission for RSV lower respiratory tract infection in infants <12 months of age.^5,21–24^ Given the disparities in definitions across these studies, it is challenging to compare results. Respiratory events were most frequently described in most studies. Only 1 study described reintubation rates and found substantially higher rates compared to our study (15.2% versus 3.5%).^5^ The study by Linssen et al^5^ reported that nearly half of the infants required reintubation for other reasons than ongoing respiratory distress after extubation and intubation injuries, including withdrawal of sedation, which we did not assess in our study. The rate of intubation injuries in our study was comparable to the study of Linssen et al,^5^ 2.6% versus 1.9% of all PICU-admitted infants. In addition, only 1 study described the incidence of pneumothorax in children admitted with RSV.^21^ The incidence of pneumothorax in our study was low, yet twice as high as that reported by Willson et al^21^ (1.4% versus 0.6%). This difference is most probably explained by the inclusion of general ward admissions to their study population. In their cohort, only 30% of infants were admitted to the PICU, and only 14% of admitted infants needed IMV. The rate of CPR in our study was comparable to the study of Willson et al,^21^ 1.1% versus 0.9% of infants.

In addition, we observed that 61.1% of infants received IMV. This is lower compared with a previous study describing RSV disease in infants admitted to the PICU in the Netherlands, which found that 71.8% of infants required IMV and showed a decreasing trend between 2003 and 2016.^5^ Global research describing the frequency of IMV in PICU-admitted infants with bronchiolitis report a great variety from 11% to 90%.^25–27^ This variety potentially reflects differences in the standard of practice, quality, resources and settings of PICUs globally.

We found that IMV is an important risk factor for the occurrence of events. IMV directly leads to the necessity for other supportive measures (eg, sedatives, intravenous or arterial catheters, urinary catheterization and laboratory measurements), which, in turn, increases the risk of occurrence of other events. For example, extravasation of infusion fluid occurred in 2.8% of infants, of whom 92% received IMV.

There are currently no data published on long-term psychological sequelae after RSV-related PICU admission. Earlier research on PTSS in parents of PICU-admitted children showed a high risk of psychological sequelae with a confirmed PTSD diagnosis in 6% to 42% of parents, which is consistent with our findings of almost 30% of parents experiencing symptoms of PTSD.^3,28,29^ It is important to note that our study solely focused on signs or symptoms of PTSD, which is not representative of the incidence of confirmed PTSD diagnosis. Research on parental and infant PTSS after an infant’s admission to the PICU is important, as it can lead to serious challenges in societal interactions, interpersonal relationships and occupational areas.^3^

Overall, we found that characteristics of PICU-admitted infants with RSV are similar to previous studies in terms of LoS,^5,25,30^ preterm births,^31^ other comorbidities and mortality.^5,25^ The age distribution was comparable with earlier research showing that the peak incidence of RSV-related PICU admissions is below 3 months of age.^2,5,25,26^ New RSV preventive interventions have shown protection against severe RSV disease up to 6 months of age.^8,13^ This underscores the potential high impact of these interventions on reducing the full burden of RSV-related PICU admissions.

A key strength of the present study is the participation of all PICUs in the Netherlands. Equally important, this study has a prospective study design that ensures real-time data collection of all RSV-related PICU admissions and occurring events. Each PICU appointed a dedicated BRICK study site collaborator to share data weekly. In addition, the research team checked all EHRs regularly to ensure complete data collection. Also, due to the increased use of virology testing during the COVID-19 pandemic, RSV testing was routinely performed in all participating hospitals resulting in a precise estimation of events occurring during RSV-related PICU admission. Finally, we included data from a standard-of-care follow-up visit after PICU admission to include parental symptoms of PTSS as a potentially important long-term event after PICU discharge.

The most important limitation of our study is that the standard of practice could differ between PICUs, which could have impacted the results. However, we aimed to describe a complete overview of the clinical burden of RSV in the Netherlands. Yet, on the other hand, data collected from EHRs might have been incomplete, leading to an incomplete overview of the course of the PICU admission. In addition, we collected data on the maximum respiratory support that was applied. We found that ≈15% of the patients received noninvasive ventilation. Noninvasive ventilation included all terms that were reported in the EHR related to noninvasive ventilation, continuous positive airway pressure or bilevel respiratory support. We did not distinguish between those terms during the data collection. In future studies, the distinguishment may help to understand possible differences in the course of PICU admission. However, for HFNC, Dutch guidelines are available. HFNC is applied in most (yet not all) pediatric departments of general hospitals. Dutch guidelines with HFNC start, stop and escalation to PICU criteria are available to ensure similar standard-of-care practices at each PICU.

Finally, this study was conducted partly during the COVID-19 pandemic, which has changed the usual RSV seasonality. This could have impacted the frequency and severity of events. Research comparing RSV burden before and during the COVID-19 pandemic showed an increase in the median age of RSV-related hospitalizations (6 vs. 9 months) and an overall decrease in PICU admissions.^32^ In the Netherlands, the median age of RSV-related hospitalizations increased from ≈2 months before the COVID-19 pandemic to ≈4 months during and after the COVID-19 pandemic.^33^ These changes may have led to an underestimation of PICU admissions and/or events because the immune system of older infants is more developed, potentially resulting in less severe disease, fewer PICU admissions and a lower incidence of occurring events. However, up until now, most studies did not find that RSV disease severity decreased during the pandemic.^34,35^ Our study did not find a difference in age distribution in infants (<1 year) admitted at the PICU for season 2021-2022 (during and after the COVID-pandemic) and season 2022-2023 (when RSV circulation started to move back to the prepandemic patterns).

This study is the first to determine the full burden of RSV infection in infants at the PICUs in the Netherlands. In summary, the burden of severe RSV disease is broader than the PICU admission alone. Unwanted, detrimental events occurred in the ≈11% of infants with RSV-related PICU admission. IMV is an obvious risk factor for the occurrence of these events. In addition, approximately one-third of parents showed signs or symptoms of PTSD. Because 75% of infants were younger than 3 months of age at PICU admission, the introduction of RSV preventive interventions, that is, maternal vaccination and/or infant immunization, into the Dutch immunization program can have the potential to significantly decrease this yet unseen RSV burden too.

## Acknowledgments

*The authors thank Peter van de Ven for his assistance with the statistical analysis of this study.*
